# Systematic Encoding and Shortening of PAC Codes

**DOI:** 10.3390/e22111301

**Published:** 2020-11-15

**Authors:** Erdal Arıkan

**Affiliations:** Elecrical-Electronics Engineering Department, Bilkent University, Ankara 06800, Turkey; arikan@ee.bilkent.edu.tr; Tel.: +(90)-312-290-1347

**Keywords:** PAC codes, polar codes, systematic encoding, code shortening

## Abstract

Polarization adjusted convolutional (PAC) codes are a class of codes that combine channel polarization with convolutional coding. PAC codes are of interest for their high performance. This paper presents a systematic encoding and shortening method for PAC codes. Systematic encoding is important for lowering the bit-error rate (BER) of PAC codes. Shortening is important for adjusting the block length of PAC codes. It is shown that systematic encoding and shortening of PAC codes can be carried out in a unified framework.

## 1. Introduction

PAC codes are a class of linear block codes designed to improve the performance of polar codes by combining channel polarization with convolutional coding [1]. It has been shown that PAC codes can perform better than polar codes [1], in some instances performing close to the theoretical limits for finite-length codes.

**Figure 1:**
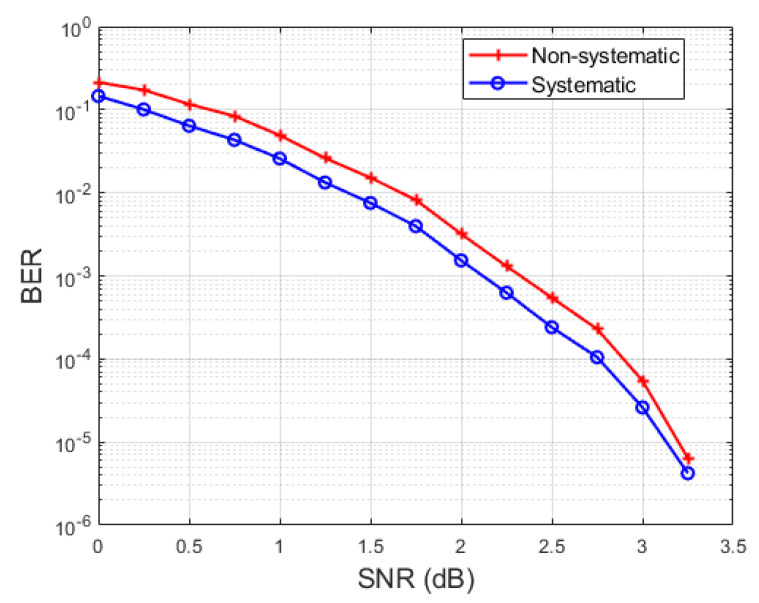
BER comparison for systematic and non-systematic PAC codes.

Given the potential of PAC codes for applications requiring extreme reliability at short block-lengths, it is of interest to investigate various aspects of PAC codes that may be important in practice. In this paper, we study systematic encoding and shortening of PAC codes. Systematic encoding is of interest mainly because it provides a better bit error rate (BER) performance compared to non-systematic encoding. Code shortening is important as a means of providing flexibility is choosing the code length. The BER advantage of systematic coding is illustrated in Figure 1 for a PAC code of length N=128 and rate R=1/2 on an additive Gaussian noise channel with binary modulation. A better BER performance is important in concatenation schemes where an outer code corrects the bit errors left over by an inner PAC code.

In Section 2, we give a definition of PAC codes and their non-systematic encoding. In Section 3, we develop a method for systematic encoding of PAC codes. In Section 4, we indicate how the systematic encoding method of Section 3 can be used for shortening PAC codes.

Throughout, we restrict attention to PAC codes over the binary field $F_{2}=\left\{0,1\right\}$. All algebraic operations are over vector spaces over $F_{2}$. $F_{2}^{N}$ will denote row vectors of length *N* over $F_{2}$ and $F_{2}^{N\times M}$ will denote matrices with *N* rows and *M* columns. For any $v=\left(v_{1},\ldots ,v_{N}\right)\in F_{2}^{N}$ and A⊂{1,2,…,N}, let $v_{A}$ denote the subvector $\left(v_{i}:i\in A\right)$. For any $G\in F_{2}^{N\times M}$, A⊂{1,2,…,N}, and B⊂{1,2,…,M}, let $G_{A,B}$ denote the matrix obtained after deleting the rows of G not in A and columns of G not in B. The notation 0 denotes a vector or matrix all of whose elements are 0 and I denotes an identity matrix.

## 2. PAC Codes

A PAC code over $F_{2}$ is a linear block code parametrized by (N,K,A,f,g) where *N* is a code block length, *K* is a code dimension, A is a data index set, $f\in F_{2}^{N-K}$ is a frozen word, and $g=\left(g_{0},g_{1},\ldots ,g_{m}\right)\in F_{2}^{m+1}$ is a convolution impulse response with $g_{0}=1$, $g_{m}=1$, with $g_{i}$ subject to design for 0<i<m. The data index set A is a subset of {1,2,…,N} with size |A|=K. The parameter (1+m) will be called the *span* of the impulse response g. The span of any impulse response g that we consider here will be bounded by the block length *N*. Sometimes, when the span cannot or need not be shown explicitly, we will write $g=(g_{0},g_{1},\ldots ,g_{N-1})$ to denote an impulse response, with the understanding that $g_{i}=0$ for *i* greater than or equal to the span of g.

An encoder for a PAC code encodes data words $d\in F_{2}^{K}$ into codewords $x\in F_{2}^{N}$ by computing a convolution followed by a polar transform. In the convolution step, a convolution input word $v\in F_{2}^{N}$ is prepared by setting $v_{A}=d$ and $v_{A^{c}}=f$, and a convolution u=v∗g is applied to v to obtain a polar transform input word $u\in F_{2}^{N}$. ($A^{c}$ denotes the complement of A in {1,2,…,N}.) In the polar transform step, the codeword $x\in F_{2}^{N}$ is obtained by computing x=uL, where $L=F^{⊗n}$ is the polar transform matrix, defined as the *n*th Kronecker power of a kernel matrix $F=\left[\begin{array}{cc} 1 & 0\\ 1 & 1 \end{array}\right]$.

The convolution step u=v∗g involves the computation

(1) $$u_{i}=\sum_{j=0}^{m}v_{i-j}g_{j},\;\mathrm{for}\;i=1,2,\cdots ,N,$$

where $v_{i-j}$ is interpreted as 0 if i−j≤0. In the following analysis, we will represent the convolution alternatively as a linear transformation u=vT where $T\in F_{2}^{N\times N}$ is an upper-triangular Toeplitz matrix of the form

(2) $$T=\left[\begin{array}{cccccccc} g_{0} & g_{1} & g_{2} & \cdots & g_{m} & 0 & \cdots & 0\\ 0 & g_{0} & g_{1} & g_{2} & \cdots & g_{m} & \cdots & \vdots \\ 0 & 0 & g_{0} & g_{1} & \ddots & \cdots & g_{m} & \vdots \\ \vdots & 0 & \ddots & \ddots & \ddots & \ddots & \cdots & \vdots \\ \vdots & \cdots & \ddots & \ddots & \ddots & \ddots & 0 & \vdots \\ \vdots & \cdots & \cdots & \ddots & 0 & g_{0} & g_{1} & g_{2}\\ \vdots & \cdots & \cdots & \cdots & 0 & 0 & g_{0} & g_{1}\\ 0 & \cdots & \cdots & \cdots & \cdots & 0 & 0 & g_{0} \end{array}\right].$$

The first row of T is determined by g and the rows that follow are shifted versions of the first row. Please note that if m=0 then T becomes the identity matrix and PAC codes contain polar codes as a special case. To exclude this possibility, PAC codes are often defined with the condition that m≥1. However, for purposes of the present paper, there is no need to place such a restriction on *m*.

The encoding operation for PAC codes can be defined more compactly by defining a generator matrix G=TL. Then, the encoder implements the mapping x=vG after preparing the vector v in the same way as above. A direct implementation of the transform x=vG, without exploiting the structure in G, has complexity $O(N^{2})$, while the two-step encoder described above has complexity O(mN) for the convolution operation and O(NlogN) for the polar transform. Since PAC codes typically have m≪N, the complexity of implementing x=vG using the triangular factorization G=TL results in significant cost savings. Below, as we develop a systematic PAC encoder, we will exploit this triangular factorization for reducing complexity.

## 3. Systematic Encoding

The above encoder for a PAC code is non-systematic in the sense that the data word d does not appear transparently as part of the codeword x. The goal in this paper is to give a systematic encoding method so that there is a subset of coordinates A such that $x_{A}=d$.

We will consider instances of the systematic encoding problem for PAC codes that are characterized by a collection of parameters (T,L,A,B,f,d) where $T\in F_{2}^{N\times N}$ is an invertible upper-triangular Toeplitz matrix, $L\in F_{2}^{N\times N}$ is the polar transform matrix (which is an invertible lower-triangular matrix), A and B are subsets of {1,2,…,N} with sizes *K* and N−K, respectively, $f\in F_{2}^{N-K}$ is a fixed vector, and $d\in F_{2}^{K}$ is a data word. Given such an instance, a systematic encoder seeks a solution to the set of equations

(3) $$x=vTL,\;v_{B}=f,\;x_{A}=d.$$

More specifically, a systematic PAC encoder seeks to determine the missing part $x_{A^{c}}$ of the codeword x subject to the conditions (3). To analyze this problem, rewrite x=vTL in terms of G=TL as

(4) $$x_{A}=v_{B}G_{B,A}+v_{B^{c}}G_{B^{c},A},\;x_{A^{c}}=v_{B}G_{B,A^{c}}+v_{B^{c}}G_{B^{c},A^{c}}$$

where $A^{c}$ and $B^{c}$ denote the complements of A and B in {1,2,…,N}, respectively. Substituting $x_{A}=d$ and $v_{B}=f$ into (4), and solving for $x_{A^{c}}$, we obtain a formal solution as

(5) $$x_{A^{c}}=d{\left(G_{B^{c},A}\right)}^{-1}G_{B^{c},A^{c}}+f\left[G_{B,A^{c}}-G_{B,A}{\left(G_{B^{c},A}\right)}^{-1}G_{B^{c},A^{c}}\right],$$

which is valid if and only if the matrix $G_{B^{c},A}$ is invertible. (Please note that $G_{B^{c},A}$ is a square matrix since the size of $B^{c}$ equals the size of A by definition.) One way to ensure that $G_{B^{c},A}$ is invertible is to choose A and B as complementary sets so that $G_{B^{c},A}$ becomes a principal submatrix $G_{A,A}$ of G. (Since G is the product of two invertible matrices, it is invertible; hence, all its principal submatrices are invertible.) We summarize this result as follows.

**Proposition** **1.** *The systematic encoding problem (3) for PAC codes has a solution whenever $B^{c}=A$, and the solution is given by*

(6) $$x_{A^{c}}=d{\left(G_{A,A}\right)}^{-1}G_{A,A^{c}}+f\left[G_{A^{c},A^{c}}-G_{A^{c},A}{\left(G_{A,A}\right)}^{-1}G_{A,A^{c}}\right].$$

Thus, in principle, we have already provided a solution to the systematic encoding problem for any PAC code. However, the complexity of solving the systematic encoding problem by computing $x_{A^{c}}$ using (6) involves $O({\left(N-K\right)}^{2})$ arithmetic operations (additions and multiplications in $F_{2}$), which may be prohibitively complex for many applications.

In the rest of this section, we develop a low-complexity systematic encoder for PAC codes under the assumption that the data index set A is chosen so that $L_{A^{c},A}=0$ is satisfied. This condition is not as restrictive as it may appear since it is satisfied by the preferred choices for the data index set A, such as when A is chosen according to a polar coding design rule or a Reed-Muller design rule [1].

For clarity, we restate the systematic encoding problem considered in the rest of this section as follows. Given a data word $d\in F_{2}^{K}$ and a data index set A for which $L_{A^{c},A}=0$, find a codeword $x\in F_{2}^{N}$ so that

(7) $$x=vTL,\;v_{A^{c}}=f,\;x_{A}=d.$$

**Proposition** **2.** *The systematic encoding problem (7) can be solved by a method consisting of the following three steps. (i) Generate an auxiliary word $c\in F_{2}^{K}$ by computing $c=d{\left(L_{A,A}\right)}^{-1}$. (ii) Compute a convolution input-output pair (v,u) so that*

(8) $$u=vT,\;u_{A}=c,\;v_{A^{c}}=f.$$

*(iii) Obtain the systematic codeword by computing the polar transform x=uL.*

**Proof.**  The second and third steps ensure that x=vTL, with $v_{A^{c}}=f$. Therefore, x is a codeword in the PAC code. Moreover, we have

$$x_{A}=u_{A}L_{A,A}+u_{A^{c}}L_{A^{c},A}=cL_{A,A}=d,$$

since $L_{A^{c},A}=0$, $u_{A}=c$, and $c=d{\left(L_{A,A}\right)}^{-1}$. Thus, $x_{A}=d$ is also satisfied, confirming that the encoding method is systematic.☐

The above systematic encoding method calculates $v_{A}$ although systematic encoding does not explicitly call for the calculation of $v_{A}$. On the other hand, the calculation of $v_{A}$ proves (implicitly) that a solution to the systematic encoding problem exists.

Next, we examine the complexity of each step of the systematic encoding method of Proposition 2.

**Proposition** **3.** *The first and third steps of the method in Proposition 2 each have complexity O(NlogN).*

**Proof.**  The third step $x=uL=uF^{⊗n}$ is a polar transform operation, which is known to have complexity O(NlogN) [2] thanks to the recursive structure of the polar transform. As for the first step, a direct computation of $c=d{\left(L_{A,A}\right)}^{-1}$ (without exploiting the special structure of the polar transform) has complexity $O(K^{2})$. A better method is to embed the calculation $c=d{\left(L_{A,A}\right)}^{-1}$ in a polar transform operation, as in systematic encoding of polar codes [3,4,5]. To that end, we recall that the inverse of the polar transform $L=F^{⊗n}$ is itself, *i.e.,*$L^{-1}=L$. This, combined with the condition that $L_{A^{c},A}=0$, implies that ${\left(L_{A,A}\right)}^{-1}=L_{A,A}$. To see this last point, note that for any two matrices $A\in F_{2}^{N\times N}$ and $B\in F_{2}^{N\times N}$,

$${\left(AB\right)}_{A,A}=A_{A,A}B_{A,A}+A_{A,A^{c}}B_{A^{c},A},$$

and let A=L and $B=L^{-1}=L$. Therefore, we have $c=d{\left(L_{A,A}\right)}^{-1}=dL_{A,A}$. Now, prepare a vector $x^{\prime }\in F_{2}^{N}$ by setting $x_{A}^{\prime }=d$ and $x_{A^{c}}^{\prime }=0$, apply a polar transform $u^{\prime }=x^{\prime }L$, and extract c from $u^{\prime }$ by setting $c=u_{A}^{\prime }$. This yields the desired result since

$$u_{A}^{\prime }=x_{A}^{\prime }L_{A,A}+x_{A^{c}}^{\prime }L_{A^{c},A}=dL_{A,A}.$$

☐

**Proposition** **4.** *The system of equations (8) in the second step of Proposition 2 can be solved by a sequential method of complexity O(mN) for a PAC code with a convolution impulse response $g=(g_{0},g_{1},\ldots ,g_{m})$ (where $g_{0}\neq 0$ by definition of PAC codes).*

**Proof.**  To develop a sequential method that solves (8), we begin by rewriting the convolution Equation (1) as follows

(9) $$u_{i}=g_{0}v_{i}+g_{1}v_{i-1}+\cdots +g_{m}v_{i-m}=v_{i}+s_{i},\;i=1,2,\ldots ,N$$

where we used $g_{0}=1$ and have defined $s_{i}=g_{1}v_{i-1}+\cdots +g_{m}v_{i-m}$ as an *i*th *feed-forward variable*. Please also note that in (9), we have used the convention that $v_{j}=0$ for j<1.

Observe that, for each 1≤i≤N, either i∈A or $i\in A^{c}$. In the former case, we obtain $u_{i}$ from the constraint $u_{A}=c$; in the latter case, we obtain $v_{i}$ from $v_{A^{c}}=f$. Given the value of one of the elements of the pair $\left(v_{i},u_{i}\right)$, the other can be found from the relation $u_{i}=v_{i}+s_{i}$. Also, observe that $s_{i}$ depends only on the knowledge of $\left(v_{1},v_{2},\ldots ,v_{i-1}\right)$. These observations suggest a sequential method for carrying out the second step of Proposition 2. The sequential method begins with i=1 with $s_{1}=0$. Either 1∈A and $\left(v_{1},u_{1}\right)=\left(c_{1},c_{1}\right)$ where $c_{1}$ is the first element of the auxiliary word c; or $1\in A^{c}$ and $\left(v_{1},u_{1}\right)=\left(f_{1},f_{1}\right)$ where $f_{1}$ is the first element of the frozen word f. In either case, we can compute $s_{2}$ before proceeding to the next step of the sequential method. In general, the *i*th step of the sequential method begins with $s_{i}$ available from the (i−1)th step and one determines the missing element of the pair $\left(v_{i},u_{i}\right)$ using the relation $u_{i}=v_{i}+s_{i}$. Thus, this method solves the system of equations (8). The method also provides a proof of existence and uniqueness of the solution.

The complexity of the sequential method given above is dominated by the complexity of calculating the feed-forward variables $\left(s_{1},s_{2},\ldots ,s_{N}\right)$. From the definition of $s_{i}$, it is clear that $s_{i}$ can be calculated using at most *m* multiplications and m−1 additions in $F_{2}$. Thus, the overall complexity is O(mN).

**Remark** **1.** *An inspection of the above proof will show that the sequential method of Proposition 4 can be used to solve the system of equations (8) for any IUT matrix T; the Toeplitz property is not essential.*

The complexity O(mN) of the sequential method of Proposition 4 corresponds to a significant savings if m≪N. If m≪N is not true, it may be worth working with the inverse of T. To discuss this, we first cite a well-known result, see e.g., [6].

**Proposition** **5.** *The class of all N-by-N IUT Toeplitz matrices form a group under matrix multiplication. Let $T\in F_{2}^{N\times N}$ be an IUT Toeplitz matrix with its first row given by $g=\left(g_{0},g_{1},\ldots ,g_{N-1}\right)\in F_{2}^{N}$. (If g has span m+1, then $g_{i}=0$ for m<i≤N−1.) Then, $T^{-1}\in F_{2}^{N\times N}$ is an IUT Toeplitz matrix with first row given by $h=\left(h_{0},h_{1},\ldots ,h_{N-1}\right)\in F_{2}^{N}$ where $h_{0}=\left(1/g_{0}\right)$ and $h_{k}=-\frac{1}{g_{0}}\sum_{i=1}^{k}g_{k-i}h_{i}$ for k=1,2,…,N−1.*

Proposition 5 allows us to recast the convolution problem (8) in an inverted form: Compute a convolution input-output pair (v,u) so that

(10) $$v=uT^{-1},\;v_{A^{c}}=f,\;u_{A}=c.$$

The inverted problem (10) has the same form as the original problem (8) with the roles of v and u reversed. Therefore, it can be solved using the same sequential method described above. There may be an advantage in solving the inverted problem if the span of the first row of $T^{-1}$ is shorter than that of T. For example, let $T\in F_{2}^{16\times 16}$ be an IUT Toeplitz matrix with first row g=(1,1,1,1,0,0,0,0,1,1,0,1,1,0,1,0), with a span of 15. The inverse $T^{-1}\in F_{2}^{16\times 16}$ is the IUT Toeplitz matrix with first row h=(1,1,0,0,1,1,0,0,0,0,1,0,0,0,0,0), which has a span of 11.

**Figure 2:**
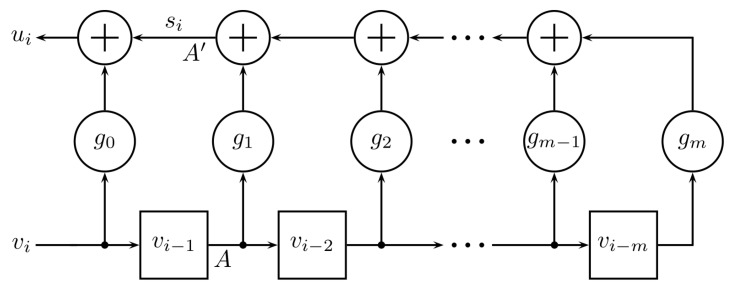
Convolution circuit.

We end this section by noting that for hardware implementations of the convolution operation in PAC encoding (both for systematic and non-systematic cases), one can use shift-register circuits that are commonly used in encoding algebraic codes. In particular, the convolution operation u=v∗g (or, equivalently the transform u=vT) can be implemented as shown in Figure 2. A version of the same circuit, with the left-most stage eliminated, generates the feed-forward variable $s_{i}$ at point $A^{\prime }$ when $v_{i-1}$ is provided as input at point *A*.

## 4. Shortening of PAC Codes

PAC codes have native lengths that are powers of two, $N=2^{n}$ for some n≥1. In many applications, it is necessary to adjust the code length to some desired value other than $2^{n}$. One method for adjusting code length is code shortening in which a portion $x_{C}$ of the codeword x is constrained to a predetermined value, say zero, and is not transmitted, effectively reducing the code length from *N* to N−|C|. A common method of code shortening for polar codes is to choose the set C so that $L_{C^{c},C}=0$ [7,8]. The systematic encoding method for PAC codes presented above can be used to implement such a shortening method.

Suppose we desire shortening of a PAC code in connection with non-systematic encoding. We partition the index set {1,2,…,N} into three disjoint sets: a data index set A, a frozen index set B, and a shortening index set C subject to the condition $L_{A\cup B,C}=0$. Then, we apply the systematic encoding method presented above to the problem

(11) $$x=vTL,\;\left(v_{A},v_{B}\right)=\left(d,f\right),\;x_{C}=0.$$

In other words, the data word d is treated as if it is part of the frozen part of the convolution input word v, and the part $x_{C}$ of the codeword is treated as if it is the data part of the codeword in a systematic PAC code.

If on the other hand, we desire to shorten a systematic PAC code, then the index set {1,2,…,N} is partitioned into a data index set A, a frozen index set B, and a shortening index set C subject to the condition $L_{B,A\cup C}=0$, and we apply the above systematic encoding method to the problem

(12) $$x=vTL,\;v_{B}=f,\;\left(x_{A},x_{C}\right)=\left(d,0\right).$$

In other words, we treat $\left(x_{C},x_{A}\right)$ as if all of it is data in a systematic PAC code.
